# Single Incision Versus Conventional Multiport Laparoscopic Sleeve Gastrectomy: Meta-Analysis and Systematic Review

**DOI:** 10.7759/cureus.46956

**Published:** 2023-10-13

**Authors:** Karim Ataya, Ayman M Bsat, Almoutuz Aljaafreh, Amir Rabih Al Ayoubi, Abdul Hafiz Al Tannir

**Affiliations:** 1 Upper Gastrointestinal Surgery, King's College Hospital, London, GBR; 2 General Surgery, American University of Beirut Medical Center, Beirut, LBN; 3 General Medicine, Faculty of Medical Sciences, Lebanese University, Beirut, LBN; 4 General Surgery, American University of Beirut Medical center, Lebanon, Beirut, LBN

**Keywords:** bariatric surgery, laparoscopic surgery, multiport, sleeve gastrectomy, single-incision

## Abstract

Laparoscopic sleeve gastrectomy (LSG) is the most widely performed bariatric surgery and has been associated with excellent outcomes and a significant reduction in obesity-related morbidity and mortality. Traditionally, this surgery is performed using five to seven trocars. However, LSG performed through a single trocar is emerging as a less invasive method of performing this surgery. This systematic review and meta-analysis compare the outcomes and complication rates of single-port versus multi-port LSG.

We searched PubMed, Medline, Scopus, and the Cochrane Library for articles published from 2008 to 2023, in accordance with the PRISMA 2020 guidelines. Data on variables such as operative time, excess weight loss, intraoperative bleeding, postoperative leak, and incisional hernia rates were collected and analyzed using a random-effects model.

Fourteen articles met the inclusion criteria and were included in the meta-analysis. No significant differences were found between the single-port LSG (SILSG) and conventional LSG (CLSG) groups in terms of operative time, rate, intraoperative complications, length of hospital stay, postoperative complications, and excess weight loss (EWL). Furthermore, single incision sleeve gastrectomy showed better satisfaction with the cosmetic score.

SILSG is a viable alternative procedure, showing comparable outcomes to multiport conventional sleeve gastrectomy, in addition, to a better cosmetic satisfaction score.

## Introduction and background

Over the past few decades, obesity rates and associated metabolic diseases have rapidly increased worldwide [1]. As our understanding of weight regulation has advanced, treating obesity has become increasingly complex. While pharmacologic treatments targeting weight regulation yield limited results, bariatric surgery remains the only effective, long-lasting solution for a significant and enduring reduction in body weight [2]. Nowadays, sleeve gastrectomy (SG) is widely recognized as the foremost bariatric technique by numerous surgeons. It has become the most performed bariatric procedure due to its simplicity and efficacy in facilitating substantial weight loss and remission of co-morbidities. The procedure was first performed in 1990 as part of a two-step surgical intervention known as biliopancreatic diversion with a duodenal switch. Subsequently, the first laparoscopic SG (LSG) procedure was conducted in 1999 and has since become the most common weight loss surgery performed in the United States [3].

Recent advancements in surgical techniques have aimed to achieve better outcomes using fewer resources. These innovations focus on minimizing pain, improving aesthetic outcomes, and shortening hospital stays, all while maintaining efficacy and minimizing complications [4]. Conventional LSG (CLSG) typically involves three to seven trocar incisions. However, it is especially suited for a single-incision approach due to its limited range of motion and the requirement to enlarge only one trocar incision to remove a specimen. This makes it an ideal candidate for single-incision LSG (SILSG) [5].

Reducing the number of abdominal incisions has the potential to bring about improvements in terms of pain and postoperative complications while mitigating the surgical stress response. This has resulted in the growth of SILSG in various domains, including bariatric surgery [6]. Based on these observations, our study aims to conduct a meta-analysis in order to compare the surgical outcomes of CLSG and SILSG in the treatment of morbid obesity.

## Review

Method

Study Design

**Figure 1:**
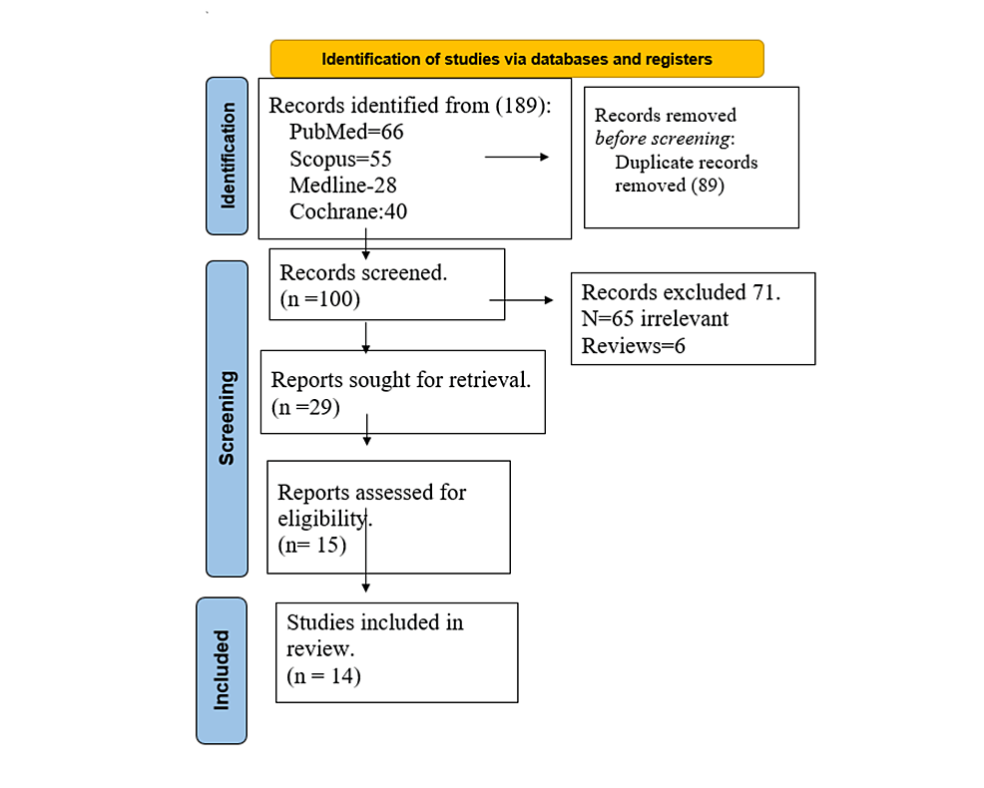
PRISMA 2020 Flow Diagram for Systematic Reviews

The present study was conducted with the utmost fidelity to a previously established methodology that had been collectively approved by all contributing authors, in conjunction with adherence to the directives outlined in the Preferred Reporting Items for Systematic Reviews and Meta-Analyses (PRISMA) guidelines (Figure 1). We conducted a comprehensive literature review to ensure a meticulous and thorough analysis.

Literature Search

This systematic review was conducted by searching multiple databases, including PubMed, Medline, Scopus, and the Cochrane Central Register, from 2008 to 2023. We used specific search terms in various combinations, including “single-incision,” “single-port,” “trans-umbilical,” “conventional,” “multiport,” and “sleeve gastrectomy.” Out of the 100 studies retrieved, 71 were excluded after screening their abstracts. The remaining 29 articles underwent full-text review, and 14 were selected for directly comparing the outcomes and complications of CLSG and SILSG.

Statistical Analysis

For categorical outcomes, we calculated the odds ratio (OR) and 95% confidence interval (CI) utilizing a random-effects model based on the Mantel-Haenszel statistical method. An OR of less than one indicated a higher prevalence of the outcome in the SILSG group. The continuous outcomes were determined by utilizing the weighted mean difference (WMD) and its 95% CI with random-effects models based on the inverse variance statistical method. A WMD of less than 0 indicated elevated values in the SILSG group. Data analysis was performed using the Cochrane Collaboration’s Review Manager (RevMan) version 5.3.

Inclusion Criteria

The inclusion criteria for this study were as follows: (1) comparative studies between CLSG and SILSG in obese patients; (2) studies published between 2008 and 2023; and (3) studies reporting original research in English. Excluded from this analysis were conference abstracts, review articles, and clinical practice guidelines.

Quality and Publication Bias Evaluation

We used the Newcastle-Ottawa Quality Assessment Scale (NOS) to evaluate non-randomized controlled trials (non-RCTs). The scale ranges from 0 to 9 stars. Studies with a score of five or higher were deemed to have sufficient methodological quality for inclusion. No RCTs met the criteria for inclusion. Two investigators, KA and AB independently rated the studies, and a final decision was made through consensus. The risk of publication bias was evaluated through the visual inspection of funnel plots.

Data Extraction

Data were extracted from each included study pertaining to demographics such as sample size for each group, age, sex, preoperative body mass index (BMI), and comorbidities. We also compiled data on perioperative outcomes, including the use of single-port devices, mean operative time, mean hospital stay, bougie diameter, conversion rate, doses of analgesics, and incidence of intraoperative and postoperative complications. Two investigators, KA and AA, carried out the data extraction process. They verified the validity of the data by reaching a consensus, with any disagreements resolved either through third-party adjudication (AAA) or by mutual agreement.

Results

Overview of Article Selection

This meta-analysis included a total of 14 studies, comprising 1,560 patients in the multiport group and 1,554 patients in the single-port group. These studies were published between 2010 and 2022 and originated from various countries, including the United States, Italy, France, Japan, Egypt, India, Qatar, Spain, Iran, and Austria. Detailed characteristics of the studies are presented in Table 1. The average body mass index (BMI) was 40 for the multiport group and 42.2 for the single-port group. 

**Table 1:** Detailed Characteristics of the Studies Included

					Patients		Female Patients		Mean Age		Mean Preop BMI	
Study ID	Type of Study	Journal	Country	Date Published	Multiport	Single port	Multiport	Single port	Multiport	Single port	Multiport	Single port
Gomberawalla et al. [7]	Matched Cohort	Obesity Surgery	USA	2014	36	36	29	34	46 (31-72)	43.33 (27-62)	43.72 (34-50)	43.06 (37-48)
Porta et al. [8]	Cohort	Obesity surgery	Italy	2017	65	65	51	53	39±2,3	36±2,9	41.01±0.4	40.09±0.3
Mauriello et al. [9]	Cohort	Obesity surgery	France	2018	187	90	106	80	52±15	45±12	53.3±3.6	45.3±2.4
Tranchart et al. [5]	Cohort	Surgical endoscopy	France	2020	512	610	377	509	40.75±2.83	42±3	44.07±1.40	40.4±2.37
Amiki et al. [10]	Cohort	Obesity surgery	Japan	2019	31	31	31	31	39.7±9.0	39.1±8.7	33.7±2.0	33.5±2.6
Alkashty et al. [11]	Case Control	International surgery journal	Egypt	2022	20	20	NA	NA	35 (25-45)	30 (20-40)	45(40-50)	42(38-46)
Lakdawala et al. [4]	Cohort	Obesity surgery	India	2011	50	50	39	40	32±9	30±8	44.75±6.25	43±6.5
Lakdawala et al. [12]	Cohort	Obesity Surgery	India	2015	300	300	150	279	35.5±7.8	35.5±9.7	39.9±5.1	39.9±5.2
Muir et al. [13]	Cohort	Obesity Surgery	USA	2016	30	32	27	31	35	32.4	46.5	42.1
Khidir et al. [14]	Cohort	Surgical innovations	Qatar	2020	220	200	121	153	34.7±16.5	33.3±10.5	48.6±8.1	43.8±5.6
Saber et al. [15]	Cohort	Surgery for obesity and related diseases	USA	2010	12	14	7	7	43.1±13.6	44.2±11	52.6±6.8	53.8±6.6
Delgado et al. [16]	Cohort	Surgical endoscopy	Spain	2012	22	20	15	15	50 (26–69)	46 (20–66)	40.6 (34.5–55.3)	40.1 (35.6-55.6)
Hosseini et al. [17]	Cohort	Iranian journal of medical scienses	Iran	2017	51	51	35	46	34.4±1.2	34.7±1.2	45.3±1.2	42.8±0.7
Sucher et al. [18]	Cohort	Journal of laparoendoscopic and advanced surgical techniques	Austria	2014	40	40	40	40	43 (24–73)	37 (19–62)	43.8 (35.0–47.8)	40.8 (35.1–45.0)

Mean Operative Time

**Figure 2:**
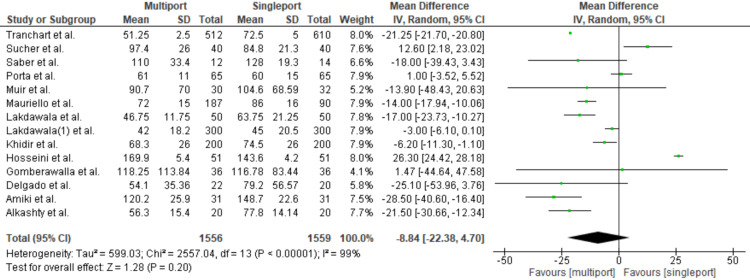
Mean Operative Time Gomberawalla et al. [7], Lakdawala et al. [4], Saber et al. [15], Porta et al. [8], Muir et al. [13], Mauriello et al. [9], Tranchart et al. [5], Amiki et al. [10], Alkashty et al. [11], Lakdawala et al. [12], Khidir et al. [14], Sucher et al. [18], Delgado et al. [16], Hosseini et al. [17]

The mean operative time ranged from 42 to 170 min in the multiport group and from 45 to 148.7 min in the single-port group. The mean difference in operative time was -8.84 [95% CI: -22.38-4.70; p = 0.2; I2: 99%]. These results indicate no statistically significant difference between the two procedures, as shown in Figure 2.

Length of Hospital Stay

**Figure 3:**
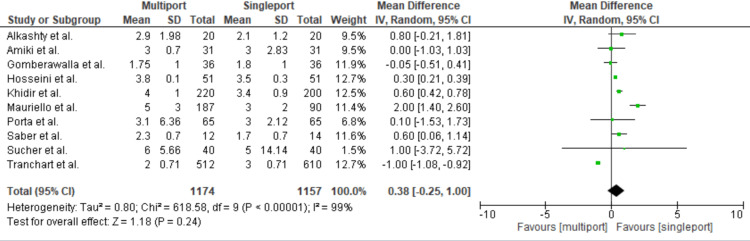
Length of Hospital Stay Gomberawalla et al. [7], Saber et al. [15], Porta et al. [8], Mauriello et al. [9], Tranchart et al. [5], Amiki et al. [10], Alkashty et al. [11], Khidir et al. [14], Sucher et al. [18], Hosseini et al. [17].

Among the 14 studies included, 10 discussed the length of hospital stay, involving a total of 1,174 patients in the multiport group and 1,157 in the single-port group. The mean length of hospital stay was comparable for both groups: it ranged from 1.75 to six days in the multiport group and from 1.7 to five days in the single-port group. The absolute mean difference was 0.38 [95% CI: -.025-1; p = 0.24; I2 = 99%]. These results demonstrate no statistically significant difference in the length of hospital stay between the two groups (Figure 3).

Post-op Leak

**Figure 4:**
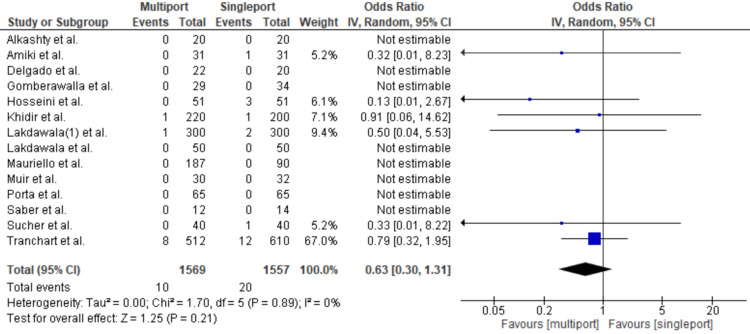
Post-op Leak Gomberawalla et al. [7], Lakdawala et al. [4], Saber et al. [15], Porta et al. [8], Muir et al. [13], Mauriello et al. [9], Tranchart et al. [5], Amiki et al. [10], Alkashty et al. [11], Lakdawala et al. [12], Khidir et al. [14], Sucher et al. [18], Delgado et al. [16], Hosseini et al. [17]

Postoperative leaks were examined in six studies. In these studies, there were 20 incidents in the single-port group and 10 in the multiport group, resulting in leak rates of 1.28% and 0.6% for patients who underwent single-port and multiport LSG, respectively. However, the difference was not statistically significant, with an OR of 0.63 [95% CI: 0.3-1.31; p = 0.21; I2 = 0%] (Figure 4).

Bleeding

**Figure 5:**
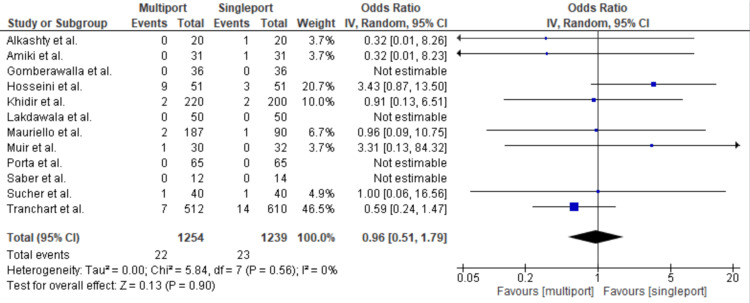
Bleeding Gomberawalla et al. [7], Lakdawala et al. [4], Saber et al. [15], Porta et al. [8], Muir et al. [13], Mauriello et al. [9], Tranchart et al. [5], Amiki et al. [10], Alkashty et al. [11], Khidir et al. [14], Sucher et al. [18], Hosseini et al. [17]

Intraoperative bleeding was almost equally distributed between the multiport and single-port groups, with 23 incidents in the single-port group compared to 22 in the multiport group, resulting in an OR of 0.96 [95%CI: 0.51-1.79; p = 0.9; I2 = 0] (Figure 5).

Surgical Site Infection

**Figure 6:**
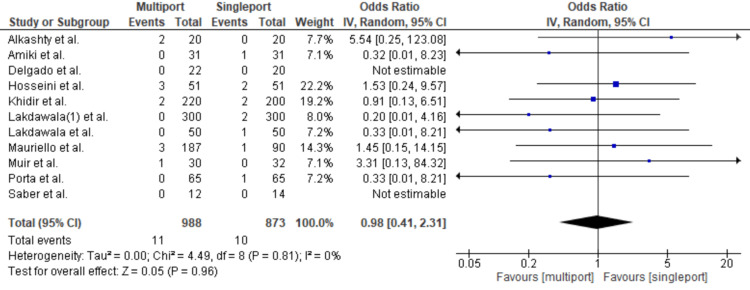
Surgical Site Infection Lakdawala et al. [4], Saber et al. [15], Porta et al. [8], Muir et al. [13], Mauriello et al. [9], Amiki et al. [10], Alkashty et al. [11], Lakdawala et al. [12], Khidir et al. [14], Delgado et al. [16], Hosseini et al. [17]

Surgical site infections occurred at nearly the same rate in both the multiport and single-port LSG groups. Analyses revealed no statistical significance, The OR was 0.98 [95% CI: 0.41-2.31; p = 0.96; I2 = 0] (Figure 6). 

Incisional Hernia

**Figure 7:**
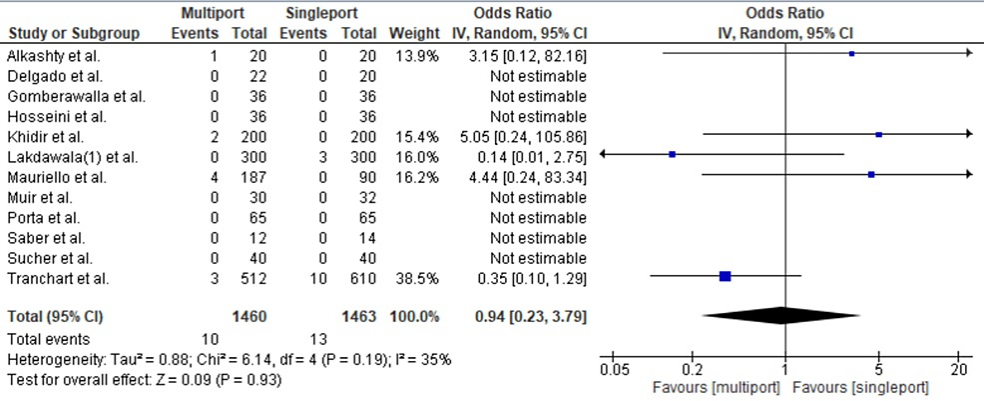
Incisional Hernia Gomberawalla et al. [7], Saber et al. [15], Porta et al. [8], Muir et al. [13], Mauriello et al. [9], Tranchart et al. [5], Alkashty et al. [11], Lakdawala et al. [12], Khidir et al. [14], Sucher et al. [18], Delgado et al. [16], Hosseini et al. [17]

Incisional hernias occurred in 13 cases in the single-port group compared with 10 cases in the multiport group, resulting in an OR of 0.94 [95% CI: 0.23-3.79, p = 0.93, I2: 35%] (Figure 7).

Excess Weight Loss at Six Months

**Figure 8:**
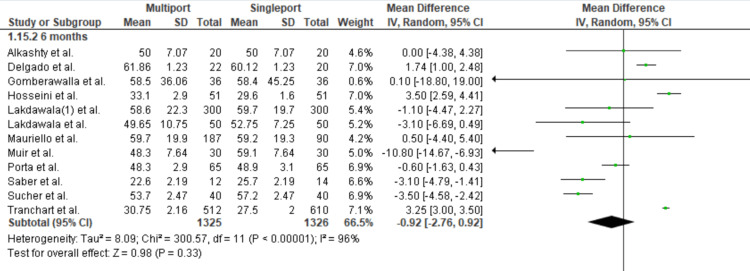
Excess Weight Loss at 6 Months Gomberawalla et al. [7], Lakdawala et al. [4], Saber et al. [15], Porta et al. [8], Muir et al. [13], Mauriello et al. [9], Tranchart et al. [5], Alkashty et al. [11], Lakdawala et al. [12], Sucher et al. [18], Delgado et al. [16], Hosseini et al. [17]

Twelve studies were included. At six months post-operation, the mean percentage of excess weight loss was 43.9% in the multiport group and 49.01% in the single-port group. Statistical analysis revealed no significant difference between the two groups, with a mean difference of -0.92 [95% CI: -2.76, 0.92; p = 0.33; I2: 96%] (Figure 8).

Cosmetic Satisfaction Score

**Figure 9:**
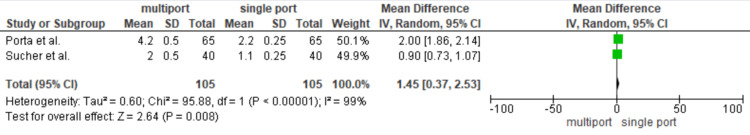
Cosmetic Satisfaction Score Porta et al. [8], Sucher et al. [18]

Two studies were included in the analysis. The cosmetic satisfaction score used a scale ranging from 1 to 5 (1 = excellent; 2 = good; 3 = fair; 4 = acceptable; 5 = poor). The analysis revealed a mean difference of 1.45 and a significant p-value, indicating superior cosmetic results in the single-incision group (Figure 9).

Gastroesophageal Reflux Disorder

**Figure 10:**
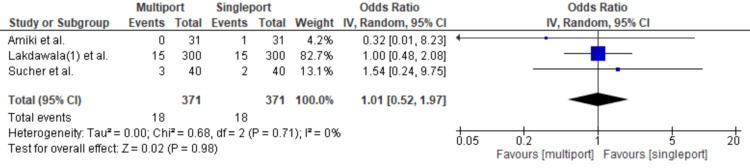
Gastroesophageal Reflux Disorder Amiki et al. [10], Lakdawala et al. [12], Sucher et al. [18]

Gastroesophageal reflux disorder was reported in only three articles. These articles revealed 18 cases in both the single-port and multiport groups, resulting in an OR of 1.01 [95% CI: 0.52-1.97; p = 0.98; I2 = 0%] (Figure 10).

Discussion

Minimally invasive surgeries offer new techniques for obese patients and present fewer side effects compared with open surgeries [19]. In 2013, LSG accounted for 43% of all bariatric surgeries in North America, 37% in Europe, 24.7% in Latin America, and 49% in the Asia-Pacific regions [20]. Consequently, significant resources and efforts are allocated to advancing this procedure and developing less invasive techniques. The emergence of single-port LSG presents a novel approach to this well-established surgery. This comprehensive evaluation and meta-analysis compare the outcomes and complication rates between traditional multi-port and single-port sleeve gastrectomy methods.

This meta-analysis found no significant differences in operative times between the two procedures. This finding aligns with a study by Delgato et al. [16], which initially indicated a statistically significant difference in operative times, with a mean of 96.5 min (60-130 min) for single-port surgery compared with 69.5 min (45-80 min) for conventional laparoscopic surgery. However, this discrepancy disappeared in the final ten cases, which suggests that initial disparities in operative time may be attributed to the learning curves of surgeons. As techniques are more widely practiced, improvements in the learning curve, as well as suturing and stapling techniques, have occurred. In contrast, Sucher et al. [18] found shorter operative times for the single-incision approach, which may be due to multiple factors. Once surgeons overcome the initial learning stage, technically challenging steps can be executed more efficiently. Furthermore, closing a single, slightly larger incision tends to be quicker than closing multiple smaller incisions, particularly in obese patients [18].

In terms of incisional hernias, we found no significant differences between the two groups. SILSG requires introducing a device to facilitate the use of instruments within the abdomen. This necessitates not only a larger skin incision but also a more substantial fascial incision, which could predispose patients to future incisional hernias. This risk was highlighted by Agaba et al. [21] who reported a port-site hernia rate of 2.9% at 30 to 36 months - a significant drawback for patients. Several factors contribute to the occurrence of port-site hernias (PSH), including both operative and patient-related elements. Most laparoscopic surgeons agree that the diameter of the cannula or port is a common cause of PSH. In SILSG, the fascial opening is larger than in conventional laparoscopy, and multiple smaller openings are combined into a single, larger one. Consequently, SILSG inherently carries a risk of PSH development [21]. However, we cannot evaluate this method in isolation; patient factors also contribute to the risk. The patient population undergoing these procedures often has a BMI above 40, adding another layer of risk alongside other factors such as diabetes and social habits.

As for cosmetic outcomes, single-incision surgery clearly offers better results and patient satisfaction. The specimen is extracted through the single-port site, which eliminates the need for additional incisions, unlike conventional LSG. Sucher et al. [18] supported this claim; at the three-month follow-up, patients evaluated their scar satisfaction using an assessment questionnaire. As expected, the SILSG group expressed greater satisfaction with their cosmetic outcomes, resulting in significantly higher scores compared to the conventional group.

## Conclusions

The findings of this systematic review indicate that SILSG is technically feasible and increasingly practiced internationally. It appears to yield clinical outcomes comparable to those of conventional multiport sleeve gastrectomy. While we caution against drawing definitive conclusions from this study, we believe there is a viable future for single-incision laparoscopic surgery as more data emerge on its safety and feasibility. We recommend further randomized controlled trials, stronger control groups, reduced heterogeneity, and extended follow-up periods to better evaluate the safety and long-term outcomes of this procedure.

## References

[1] NCD Risk Factor Collaboration (2016). Trends in adult body-mass index in 200 countries from 1975 to 2014: a pooled analysis of 1698 population-based measurement studies with 19·2 million participants. Lancet.

[2] Albaugh VL, Abumrad NN (2018). Surgical treatment of obesity. F1000Res.

[3] Seeras K, Sankararaman S, Lopez PP (2023). Sleeve gastrectomy. http://www.ncbi.nlm.nih.gov/books/NBK519035/.

[4] Lakdawala MA, Muda NH, Goel S, Bhasker A (2011). Single-incision sleeve gastrectomy versus conventional laparoscopic sleeve gastrectomy--a randomised pilot study. Obes Surg.

[5] Tranchart H, Rebibo L, Gaillard M, Dhahri A, Lainas P, Regimbeau JM, Dagher I (2020). Short-term outcomes of single-port versus conventional laparoscopic sleeve gastrectomy: a propensity score matched analysis. Surg Endosc.

[6] Morales-Conde S, Del Agua IA, Moreno AB, Macías MS (2017). Postoperative pain after conventional laparoscopic versus single-port sleeve gastrectomy: a prospective, randomized, controlled pilot study. Surg Obes Relat Dis.

[7] Gomberawalla A, Salamat A, Lutfi R (2014). Outcome analysis of single incision vs traditional multiport sleeve gastrectomy: a matched cohort study. Obes Surg.

[8] Porta A, Aiolfi A, Musolino C, Antonini I, Zappa MA (2017). Prospective comparison and quality of life for single-incision and conventional laparoscopic sleeve gastrectomy in a series of morbidly obese patients. Obes Surg.

[9] Mauriello C, Chouillard E, d'alessandro A, Marte G, Papadimitriou A, Chahine E, Kassir R (2018). Retrospective comparison of single-port sleeve gastrectomy versus three-port laparoscopic sleeve gastrectomy: a propensity score adjustment analysis. Obes Surg.

[10] Amiki M, Seki Y, Kasama K, Pachimatla S, Kitagawa M, Umezawa A, Kurokawa Y (2019). Reduced-port sleeve gastrectomy for morbidly obese japanese patients: a retrospective case-matched study. Obes Surg.

[11] Alkashty ME, Bakr AA, Nashed GA, Fahmy MH, Elward AS (2022). Comparison of short-term outcomes between multi-port and single-port sleeve gastrectomy: a prospective study. Int Surg J.

[12] Lakdawala M, Agarwal A, Dhar S, Dhulla N, Remedios C, Bhasker AG (2015). Single-incision sleeve gastrectomy versus laparoscopic sleeve gastrectomy. A 2-year comparative analysis of 600 patients. Obes Surg.

[13] Muir KB, Rice WV (2016). Weight-loss outcomes of SPIDER(®) sleeve gastrectomy at 6 months compared to traditional laparoscopic technique. Surg Endosc.

[14] Khidir N, Gagner M, El Matbouly M (2020). Single-port sleeve gastrectomy compared with conventional laparoscopic sleeve gastrectomy: 5-year follow-up of weight loss, comorbidity resolution, and cost. Surg Innov.

[15] Saber AA, El-Ghazaly TH, Dewoolkar AV, Slayton SA (2010). Single-incision laparoscopic sleeve gastrectomy versus conventional multiport laparoscopic sleeve gastrectomy: technical considerations and strategic modifications. Surg Obes Relat Dis.

[16] Delgado S, Ibarzabal A, Adelsdorfer C, Adelsdorfer W, Corcelles R, Momblán D, Lacy AM (2012). Transumbilical single-port sleeve gastrectomy: initial experience and comparative study. Surg Endosc.

[17] Hosseini SV, Hosseini SA, Al-Hurry AM, Khazraei H, Ganji F, Sadeghi F (2017). Comparison of early results and complications between multi-and single-port sleeve gastrectomy: a randomized clinical study. Iran J Med Sci.

[18] Sucher R, Resch T, Mohr E, Perathoner A, Biebl M, Pratschke J, Mittermair R (2014). Single-incision laparoscopic sleeve gastrectomy versus multiport laparoscopic sleeve gastrectomy: analysis of 80 cases in a single center. J Laparoendosc Adv Surg Tech A.

[19] Miller MR, Choban PS (2011). Surgical management of obesity: current state of procedure evolution and strategies to optimize outcomes. Nutr Clin Pract.

[20] Angrisani L, Santonicola A, Iovino P, Formisano G, Buchwald H, Scopinaro N (2015). Bariatric surgery worldwide 2013. Obes Surg.

[21] Agaba EA, Rainville H, Ikedilo O, Vemulapali P (2014). Incidence of port-site incisional hernia after single-incision laparoscopic surgery. JSLS.

